# Distinct Metabolism of Bone Marrow Adipocytes and their Role in Bone Metastasis

**DOI:** 10.3389/fendo.2022.902033

**Published:** 2022-06-21

**Authors:** Yixuan Li, Shan Cao, Anastasia Gaculenko, Yifan Zhan, Aline Bozec, Xiaoxiang Chen

**Affiliations:** ^1^ Department of Rheumatology, Renji Hospital, Shanghai Jiao Tong University School of Medicine, Shanghai, China; ^2^ Department of Internal Medicine 3, Rheumatology and Immunology, Universitätsklinikum Erlangen, Friedrich-Alexander-Universität (FAU) Erlangen-Nürnberg, Erlangen, Germany; ^3^ Drug Discovery, Shanghai Huaota Biopharmaceutical Co. Ltd., Shanghai, China

**Keywords:** bone marrow adipocytes, lineage-tracing, metabolism, bone metastasis, multi-omic analysis

## Abstract

Bone marrow adipocytes (BMAs) represent 10% of the total fat mass of the human body and serve as an energy reservoir for the skeletal niche. They function as an endocrine organ by actively secreting fatty acids, cytokines, and adipokines. The volume of BMAs increases along with age, osteoporosis and/or obesity. With the rapid development of multi-omic analysis and the advance in *in vivo* imaging technology, further distinct characteristics and functions of BMAs have been revealed. There is accumulating evidence that BMAs are metabolically, biologically and functionally unique from white, brown, beige and pink adipocytes. Bone metastatic disease is an uncurable complication in cancer patients, where primary cancer cells spread from their original site into the bone marrow. Recent publications have highlighted those BMAs could also serve as a rich lipid source of fatty acids that can be utilized by the cancer cells during bone metastasis, particularly for breast, prostate, lung, ovarian and pancreatic cancer as well as melanoma. In this review, we summarize the novel progressions in BMAs metabolism, especially with multi-omic analysis and *in vivo* imaging technology. We also update the metabolic role of BMAs in bone metastasis, and their potential new avenues for diagnosis and therapies against metastatic cancers.

## Introduction

Several types of cancer cells evade clinical treatment by niching into the bone, such as cancer of the breat, prostate, lung and melanoma. Additionally, the bone marrow is a depot for fat-storing adipocytes, which poses a highly dynamic and metabolically active organ. Therefore, the role of bone marrow adipocytes (BMAs) and their effect on niching tumor cells and subsequent tumor growth are of clinical interest. Several studies have demonstrated that BMAs could function as an energy reservoir for the skeletal niche and serve as an endocrine organ secreting fatty acids, cytokines, and adipokines, supporting cancer cells to niche and grow within the bone marrow microenvironment. Because BMAs are deeply embedded in the bone marrow niche, the isolation of sufficient numbers of BMAs from rodent or human bone marrow remains a challenge. In contrast to white adipose tissues embedded in a matrix consisting of collagen, fibronectin and laminin, BMAs are distributed as single cells or patches in the bone marrow (1). Moreover, their large size and buoyancy do not facilitate their isolation by pelleting or cell sorting. The previous histomorphometric methods could only supply limited descriptions about these cells at the cellular level, such as alterations in structure and organelles, but no further information about molecular changes. Therefore, the characterization of BMA functions in bone metastasis is currently challenging.

Nevertheless, these gaps in understanding the underlying mechanisms have been largely filled in the recent decade due to the rapid development of multi-omic analysis and *in vivo* imaging. Technologies as RNA-seq, single-cell RNA-seq (scRNA-seq), gas chromatography-mass spectrometry (GC-MS), or liquid chromatography-mass spectrometry (LC-MS), gave insights into the transcriptomic, proteomic, and metabolic depth of BMAs. Using lineage tracing, fate mapping technologies and positron emission tomography-computed tomography (PET/CT) with 18F-fluorodeoxyglucose ([^18^F]FDG), distinct characteristics and functions of BMAs have been revealed in both rodents and humans. Recent findings demonstrate the importance of BMAs as metabolically, biologically, and functionally unique adipocyte subsets distinct from white, brown, beige and pink adipocytes. Here, we summarize the novel research on BMAs, especially the unique metabolic specificity and their potential function in supporting bone metastasis.

## Anatomy

In the human body, BMAs are mainly located in the arms, legs, and sternum but rarely in the clavicle, ribs, and vertebrae (2). Meanwhile BMAs can also be observed in caudal (tail) vertebrae but not in thoracic or lumbar vertebrae (3). Interestingly, in human adults, BMAs represent around 10% of the total adipose tissue mass (4). By the age of 25 years, around 70% of the bone marrow volume in healthy adults is filled with BMAs (5). These cells can mainly be found in long bones in early adulthood. However, around 60 years of age and over, BMAs display age-associated increases in the axial skeleton (6). In long bones, BMAs dwell among the trabecular bone of the epiphysis and metaphysis or close to the endosteal surface of the diaphysis (7). BMAs have been historically overlooked and were considered “fillers” of the inert space for a long time (7). However, with the increasing interest in immunometabolism, they have raised more attention, especially for their distinct metabolic process and the consequent functional alterations.

As early as 1976, Tavassoli has discovered two distinct populations of BMAs in the bone marrow: the performic acid-Schiff (PFAS) – positively stained BMAs in red marrow and the PFAS-negatively stained BMAs in yellow marrow. The two populations also respond differently during the expansion of hematopoiesis (8). In 2015, using the osmium tetroxide staining, Scheller et al. defined for the first time regulated bone marrow adipocytes (rBMAs or red marrow BMAs) and constitutive bone marrow adipocytes (cBMAs or yellow marrow BMAs) (9). cBMAs develop after birth, are large in size and localized in close proximity to each other with a lack of hematopoietic cells in between (10). Their lipid storages mainly contain unsaturated fatty acids. In contrast, the smaller rBMAs develop throughout life and contain mostly saturated fatty acids. In steady state, rBMAs are single cells distributed within areas of active hematopoiesis.

Several environmental factors have been reported to promote the dynamic changes of BMAs. In several publications and our own data, high-calorie feeding such as high-fat diet increases number and size of BMAs. Here, mostly rBMAs localized in the metaphysis of the proximal tibia expand as response to changes in diet and diseases (11, 12). The special location of fat induced expansion of BMAs was confirmed in humans suffering from obesity, diabetes and/or osteoporosis (13, 14). In mice, irradiation and activation of the adipocyte differentiation pathway Peroxisome proliferator-activated receptor gamma (PPARγ) leads to a steady induction of BMA expansion (15). Additionally, expansion of BMAs can be observed in murine models of aging or ovariectomy-induced osteoporosis similar to the observations in patients (16, 17). Intriguingly, caloric deprivation in patients also increases the number of BMAs with gender difference regarding their localization, in L4 vertebra for men and at the femoral metaphysis for women (13). In addition, the psychiatric disease anorexia nervosa paradoxically leads to expanded bone marrow adipose tissue, while other fat depots in the body are reduced in size (18).

## Origin

The origin of BMAs has been investigated for decades and is still updating thanks to the development of advanced technologies. In 1976, BMAs were first depicted as derived from a unique progenitor distinct from white adipocytes (19). Nevertheless, due to the limited technical conditions, the differences between BMAs and their extramedullary counterpart were only described roughly according to their morphology. Nowadays, lineage tracing reporter mice and the large-scale, single-cell RNA-sequencing (scRNA-seq) have helped to delineate their features in more details.

BMAs are thought to be derived from Sca1^+^ CD45^−^ CD31^−^ or LepR^+^ CD45^−^ CD31^−^ mesenchymal stem cells (MSCs) in the bone marrow (20, 21). Using *in vivo* cell lineage tracing of the dTomato^+^ in *Vav1-Cre: mT/mG* mice, BMAs are further confirmed to be originated from MSCs but not hematopoietic stem cells (HSCs) (22, 23). Pathway enrichment analysis also displayed that BMAs are closer to bone marrow mesenchymal stem cells (BMSCs) than to white adipocytes (24). Moreover, in contrast to brown adipocytes, BMAs are all dTomato^-^ in *Myf5-Cre: mT/mG* mice (25, 26). This indicates that BMAs do not share the same progenitors as brown adipocytes. Further studies demonstrated that BMA progenitors can express Prx1 and Osx1, two markers labelling mesenchymal-osteogenic cells, while white and brown adipocytes cannot be traced in Osx1-Cre reporter mice (27, 28). In another study, using the lineage tracing of *Adipoq* ^Cre+/mTmG+^ and *UCP1* ^Cre+/mTmG+^ mice, BMAs were demonstrated to not express *UCP1* during development or upon the stimulation of β3-adrenergic agonist CL316,243 (29). These results indicate that BMAs derive from a mesenchymal-osteogenic lineage, and are genetically distinct from white, beige or brown adipocytes. Most recently, with the help of *Adipoq* ^Cre+/DTA+/mTmG+^ triple mutant mice, a defined cluster of adiponectin-negative stromal progenitors has been shown in the bone marrow of fat-free mice. This population was able to differentiate into ectopic BMAs with age and metabolic diseases. These BMAs have increased lipid storage and are not thermogenic as they are unresponsive to cold stress or β3-adrenergic stimulation (30). Despite that adiponectin is an essential adipocyte specific cytokine, the discovery of adiponectin-independent BMA subsets allows to speculate that further origins of BMAs remain to be revealed. Indeed, Zhong et al. have already defined a new population in the bone marrow from their scRNA-seq data, termed marrow adipogenic lineage precursors (MALPs) (31). This subpopulation expresses typical adipocyte markers as *Pparg, Cebpa, Adipoq, Apoe*, and *Lpl*, but not *Plin1*, thus containing no lipid droplets. They are not proliferative precursors for adipocytes but are essential for maintaining marrow vasculature and promoting pathologic bone loss in a RANKL-dependent manner (32, 33). Together, these data have vastly enriched the framework between MSCs and mature adipocytes, bringing more directions for future investigations.

**Figure 1:**
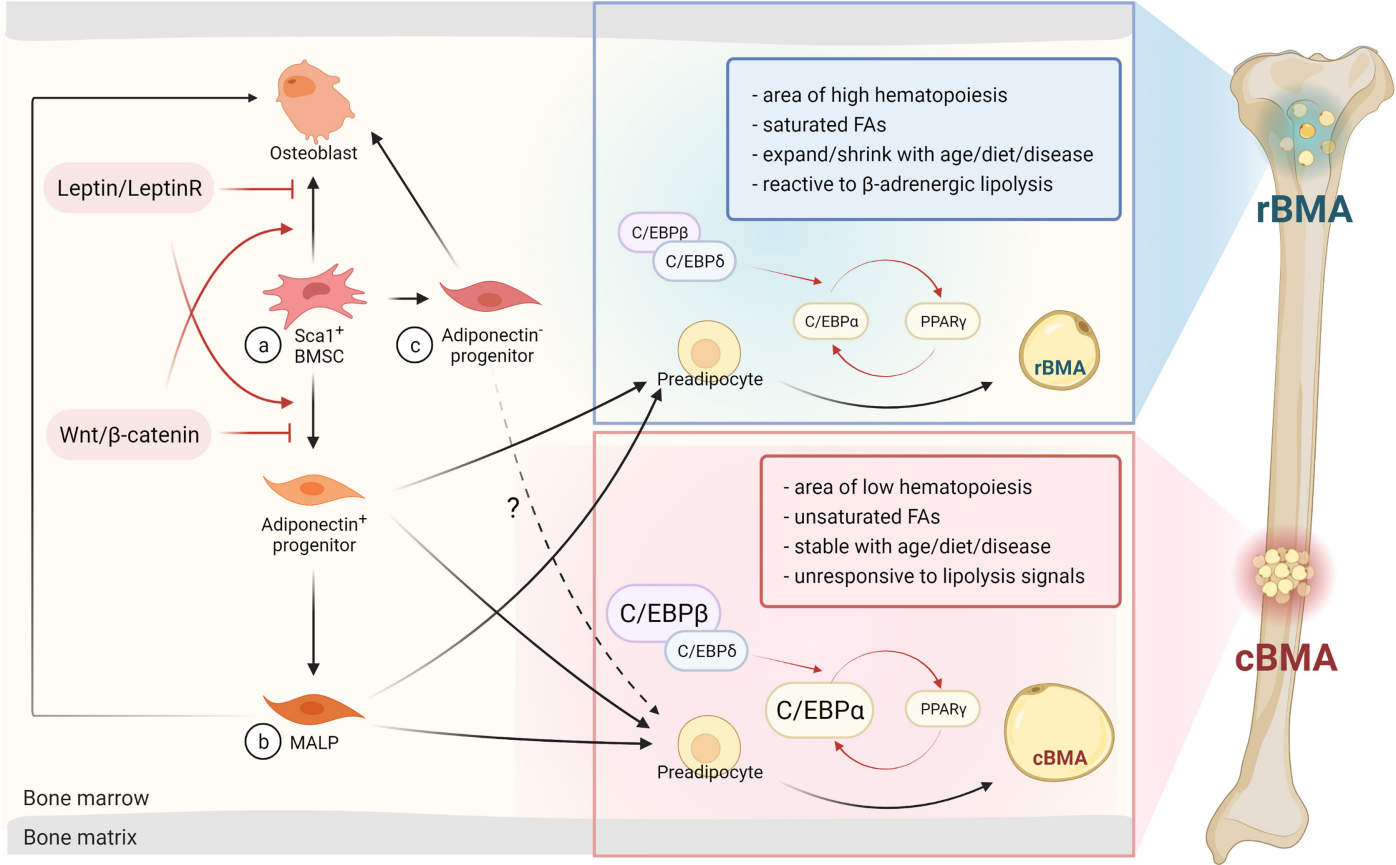
BMAs arise from BMSCs and can differentiate *via* osteogenic or adipogenic progenitors into rBMAs or cBMAs. **(A)** BMAs or osteoblasts originate from Sca1^+^ BMSCs modulated by the Leptin/LeptinR or Wnt/β-catenin signaling pathways (20–23, 35, 36). **(B)** MALPs are a newly defined primarily adipogenic sub-population that arises from adiponectin^+^ progenitors. Factors like acute injury and aging can trigger osteogenic differentiation of MALPs (30, 31). **(C)** Adiponectin^-^ progenitors are predominantly of the osteogenic lineage, but are also able to differentiate into BMAs in metabolic disorders or in aging adults. This population elicits similar properties as cBMAs (30). BMSC, bone mesenchymal stem cells; rBMA, regulated bone marrow adipocyte; cBMA, constitutive bone marrow adipocyte; MALP, marrow adipogenic lineage precursor; C/EBP, CCAAT/enhancer-binding protein; FA, fatty acid; PPARγ, peroxisome proliferator-activated receptor gamma. Red arrows indicate transcription factors and signaling pathways. Dark arrows represent the consecutive stage of differentiation. The dashed arrow emphasizes similarities in cBMAs and adiponectin^-^ progenitor-derived BMAs. Designed by Biorender.

The differentiation fate of BMAs from MSCs is also rigorously regulated by transcriptional cascades (34) (**Figure 1**). The transcription factors CCAAT/enhancer-binding protein CEBPβ and δ are induced primarily during early adipogenesis. Then they activate the expression of two critical adipogenic transcription factors: PPARγ and CEBPα (37). Expression of *Cepba* and *Cebpb* are selectively elevated in cBMAs of rats compared to rBMAs and subcutaneous white adipocytes (9). In addition, the tug-of-war between adipocytes and osteoblast differentiation in the bone marrow is also determined by many pathways such as Wnt/β-catenin and Leptin/LepR signaling. Wnt/β-catenin signaling promotes a cell fate shift from adipocytes to pre-osteoblasts (35, 36), while Leptin/LepR signaling facilitates adipogenesis and inhibits osteogenesis (21) (**Figure 1**).

In addition to the rigorous regulation of transcription cascades of BMA differentiation, the dynamic and complex bone marrow microenvironment could also be an essential contributor. Osteocyte-derived sclerostin, a glycoprotein encoded by *SOst* gene, could promote the expression of the adipogenic transcription factors *Pparγ* and *Cebpα* in primary MSCs from both humans and mice *in vitro*. As a consequence, the adipocyte differentiation *via* inhibition of the canonical Wnt signaling pathway was enhanced. *In vivo* studies also found decreased BMA formation in both sclerostin knock-out mouse models and wild-type mice treated with a sclerostin neutralizing antibody (38–40). These studies demonstrated a role for *SOst* and osteocyte-derived sclerostin in regulating fate determination of BMA progenitors. Bone morphogenetic proteins (BMPs) could also promote adipogenesis by promoting the expression of *Pparγ* and *Cebpα* (41). Bajaj and colleagues reported that *BMP4* was highly expressed and secreted especially by T cells and stromal cells in response to irradiation. Thereby, the adipogenic commitment of the M2-10B4 cell line and primary murine MSCs were promoted. This could probably be one of the causes of marrow adipogenesis post-myelosuppression (42). These extrinsic factors generated by the marrow microenvironment may contribute to the distinct metabolic features and function of BMAs compared to white adipocytes, even though much still remains to be further elucidated.

## Metabolic Features

Recent technologies have also unveiled numerous novel metabolic features of BMAs. Attané and colleagues compared the proteomic and lipidomic features of BMAs with subcutaneous fat tissue and concluded that BMAs display a distinct lipid metabolism contrary to classical white adipocytes (45). Pathway enrichment in proteomic results displayed elevated cholesterol metabolism in BMAs, which was further confirmed by a 1.5-fold increase in free cholesterol content and decreased lipolytic activity in BMAs. Moreover, more sphingosine, fewer ceramides and sphingomyelin were observed in the lipid profiles of BMAs compared to subcutaneous white adipocytes. The monoacylglycerol lipase (MGLL) expression is reduced with monoacylglycerol (MG) species elevated in BMAs, implying on an impaired MG hydrolysis compared to subcutaneous fat tissues. The altered lipid metabolism is also corroborated in another study, delineating that human BMAs possess distinct gene expression profiles, especially in regulating lipid metabolism, stemness genes, and browning pathways compared to subcutaneous adipose tissue (24). The overall steady state molecular signature of BMAs was described more comparable to brown adipocytes. In contrast, BMA expansion by aging or diabetes leads to a steady energy storing, white adipocyte-resembling metabolic signature (46). Scheller et al. also reported the diminished lipid hydrolysis in BMAs compared to white adipose tissue in response to β-adrenergic stimulation, mainly in distal regions (47). Transcriptomic analysis in rabbits also revealed decreased glycerol content, insulin resistance, reduced lipid synthesis, and transport, decreased fatty acid metabolism, and decreased thermoregulation in BMAs compared to white adipocytes. Reduction in fatty acid β-oxidation (FAO) and oxidative phosphorylation were also found in BMAs (29).

The glucose metabolism in BMAs and their role in systemic glucose homeostasis are also unique. The transcriptome analysis in rabbits and humans both revealed an altered glucose metabolism and diminished insulin responsiveness in BMAs compared to white adipocytes, while markers of brown or beige adipocytes were enriched. Using PET/CT and [^18^F] FDG, it was recently demonstrated that BMAs possess high basal glucose uptake both in rodents and humans but are unresponsive to insulin, cold exposure and glucocorticoids (2). However, in another clinical trial, Tam et al. as well used PET/CT and [^18^F] FDG to characterize the glucose uptake (GU) in human femoral and vertebral BMAs, found that insulin enhances GU in human femoral BMAs (48). These two conflicting results indicate that different species (rodents vs. human) and different sites (distal tibia BMAs vs. femur BMAs) vary significantly in BMA metabolism.

Metabolic programming also plays an important role in regulating BMA differentiation. BMA progenitors display higher insulin-dependent glucose utilization, enhanced capacity for oxidative phosphorylation (OXPHOS) and lipid storage, while osteoblast progenitors exhibit diminished insulin signaling, glycolysis-prone energy production, and reduced lipid storage (49). Moreover, metabolic changes in diseases such as obesity, diabetes and anorexia nervosa could also affect the formation of BMAs. Dyslipidemia caused by overnutrition in obesity facilitates BMA expansion and BMAs could then buffer extra energy in the form of triglycerides (50). The impaired lipid metabolism of type 2 diabetes (T2D) is characterized by the elevated low-density lipoprotein (LDL) cholesterol and free fatty acids, high concentration of plasma triglyceride and decreased high-density lipoprotein (HDL) cholesterol (51). This kind of hyperlipidemia could probably be associated with the enhanced adiposity of the bone marrow, for fatty acids could bind and activate PPARγ (52). In addition, hyperglycemia could induce expression of PPARγ by activating PI3K/Akt pathway and therefore enhance the adipogenicity of MSCs (53). The production of reactive oxygen species (ROS) resulting from the increased glucose levels in T2D could also promote the expression of genes associated with adipogenesis (54, 55). Starvation or fasting caused by anorexia nervosa also leads to hyperlipidemia (56), which could probably partly explain the expanded BMAs mentioned before. Collectively, BMA formation seems to be much closer to serum lipid levels than the type of diseases.

The number of BMAs and osteoblasts might be reciprocal, since they are competing for the same original stem cells. However, BMAs could also interfere with skeletal homeostasis and bone remodeling *via* its metabolic activities (57). The maintenance of bone mass depends on the dynamic and precise coordination of osteoclast-dominated bone resorption and osteoblast-mediated bone formation (58, 59). Studies in rats and dogs indicated reduced osteoblast activity, osteoclast numbers and increased bone loss at sites with higher BMA numbers (57). As osteoblasts are highly dependent on fatty acids for their glycolytic energy production, taking up to 80%, intact BMAs could be of importance for osteoblast function (60, 61). Moreover, Fatty acids, cholesterol, phospholipids and endogenous metabolites have been proven to regulate numerous signaling pathways mediating the proliferation and function of local osteoclasts and osteoblasts (62). Besides energy resources, BMAs may also protect osteoblasts from lipotoxicity (63). Other studies have shown the existence of BMA-derived exosomes filled with adipogenic factors and anti-osteoblastic miRNAs that are able to alter osteoblast function (64). Nevertheless, the role of BMA metabolism on bone cell survival and function remains poorly understood, and would require further investigation.

## Metabolic Role in Bone Metastasis

Bone is one of the main organs for metastasis by various tumors. Hernandez et al. have retrospectively analyzed the real world electronic medical record data from oncology practices in the US and estimated the cumulative incidence of bone metastasis among patients with various solid tumors (65). The prostate cohort had the highest risk of bone metastasis with an incidence of 18.0% at one year, 20.4% at two years, 24.5% at five years, and 29.2% at ten years followed by lung (10.4-12.9%), renal (5.8-9.9%), breast (3.4-8.1%), gastrointestinal (2.3-3.6%), malignant melanoma (1.6-3.0%) and other tumors. In addition, the incidence of bone metastasis increased by the stage at diagnosis in all studied tumors. Another retrospective population-based study using data (2010-2015) from Surveillance, Epidemiology, and End Results (SEER) program, has reported that 5.7% of cancer patients suffer from bone metastasis (66). The third most vital factor for cancer is obesity, while smoking and infection pose number 1 and 2, respectively (67). Indeed, approximately 40% of cancers are associated with the excess of body weight (68). Researchers were able to show, that the risk of metastasis formation in obese breast cancer patients is increased by 46% (69). Overall, the link between the expansion of adipose tissue and metastasis formation has become evident in the recent decade, while the mechanism underlying bone metastases and BMAs remains unclear to date.

**Figure 2:**
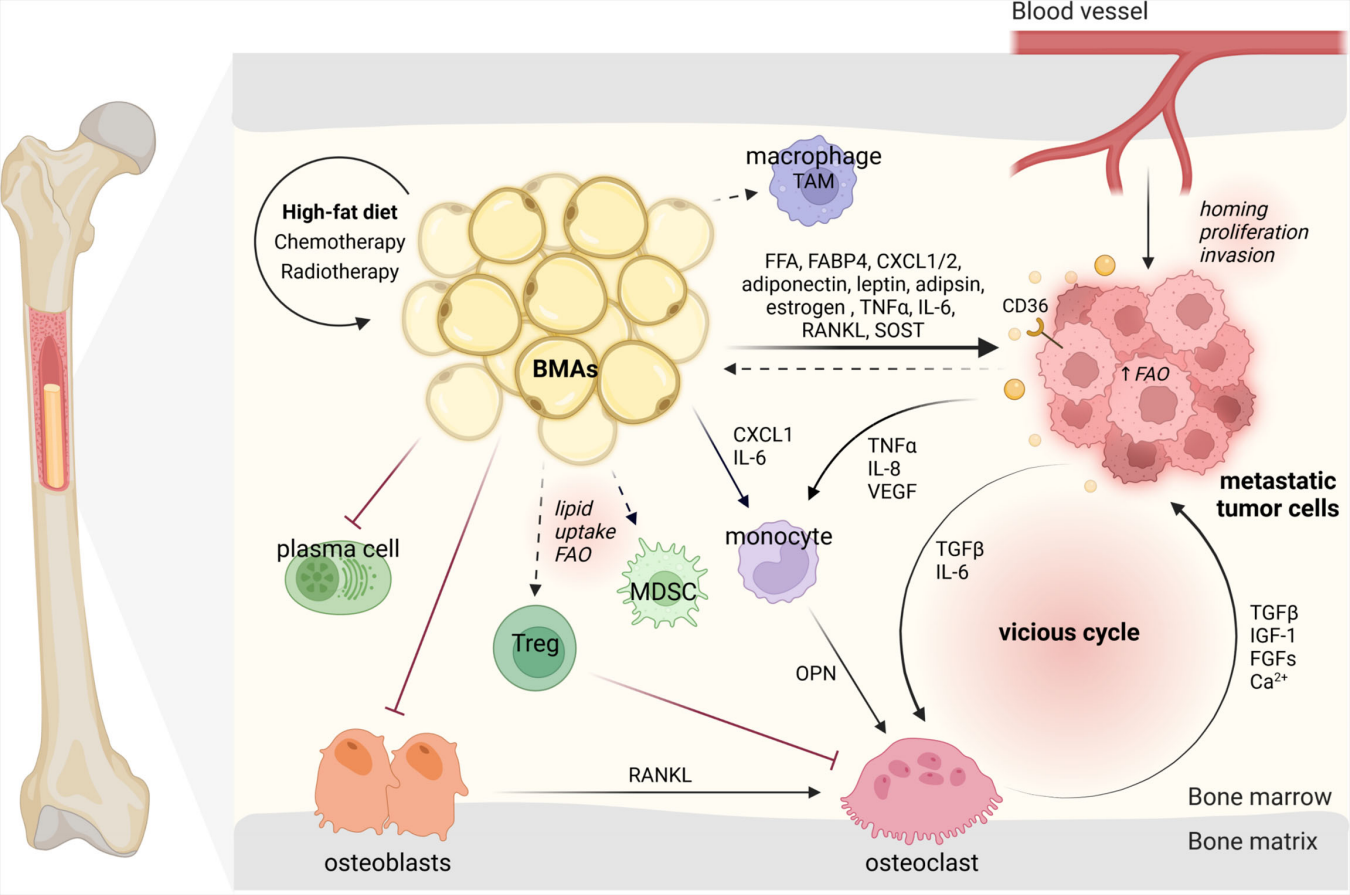
BMAs modulate their surrounding microenvironment and interact with niching tumor cells and bone marrow-resident cells. BMAs, bone marrow adipocytes; TAM, tumor-associated macrophage; FFA, free fatty acid; FABP4, fatty acid-binding protein 4; CXCL1/2, C-X-C motif ligand 1/2; FAO, fatty acid oxidation; TNFα, tumor necrosis factor alpha; VEGF, vascular endothelial growth factor; TGFβ, transforming growth factor beta; IGF-1, insulin-like growth factor-1; FGFs, fibroblast growth factors; OPN, osteopontin; RANKL, receptor activator of NF-κB ligand; Tregs, regulatory T cells. The dark arrows indicate relationships, while dashed arrows represent potential links (43, 44). Designed by Biorender.

The novel findings in the metabolism of BMAs could be of vital importance for the understanding of tumor cell niching and growth in the bone marrow (**Figure 2**). In our previous work, we were able to observe that increased numbers of BMAs lead to accelerated melanoma tumor growth in the bone marrow and can be abrogated by inhibiting the adipocyte differentiation *via* PPARγ with the pharmacological compound bisphenol-A-diglycidylether (BADGE) (11, 12). Further experiments demonstrated that increased adipogenic differentiation of pre-adipocytes boosted by melanoma cell-derived factors led to the increase of BMAs at the early stage of bone metastasis, which further favored the tumor cells to niche and proliferate (70). Moreover, it is known that upregulated number of BMAs after chemotherapy and radiotherapy can correlate with tumor evasion (71). Finally, the facts that bone metastasis occurs preferentially in older people who have a higher portion of adipocytes in the bone marrow, and that BMAs rapidly expand (9-32%) in tumor patients over one year (72) further confirmed the close connection between BMAs and bone metastasis. As a result, the involvement of BMAs in the “vicious cycle” of tumor cells and bone cells seems to accelerate tumor growth. However, recent starving therapies have obtained a gratified result in eliciting an anti-tumor response (73, 74), while BMAs were also observed elevated in these fasting-like conditions. The ambiguous results may depend on the type of the tumor cells and stage of the disease, or the individual state of BMA subsets.

In general, tumor cells metastasize to rBMAs-enriched regions (proximal femur, hip, and lumbar spine) which contain smaller and less stable adipocytes (75). This preference may be directly connected to the distribution of blood vessels allowing distinct distribution of nutrition and oxygen concentration (76). The mechanisms underlying the pro-tumor effects of BMAs have attracted considerable attention. Many publications have discussed the importance of adipokines released by BMAs in bone metastasis, such as adiponectin (71), leptin (77), adipsin (78) and estrogen (79). Others have focused on the pro-inflammatory cytokines released by BMAs like TNFα, IL-6 and RANKL or target on BMAs like sclerostin (75, 80). But the metabolic functions of BMAs during bone metastasis have been less reviewed.

BMAs are a direct power station for tumor cells *via* lipolysis and lipid transfer (81). Using the vibrational spectroscopic technique-Fourier transform infrared (FTIR) microspectroscopy, Ehsan and his colleagues demonstrated that prostate cancer cells take up isotopically labeled FA [deuterated palmitic acid (D(31)-PA)] from human MSC-derived adipocytes (82). Furthermore, they also observed the lipid uptake of prostate cancer cells from nearby BMAs in the bone metastases specimens from patients, providing direct evidence of BMAs inducing tumor growth (83). BMAs could also shape tumor cell metabolism, contributing to their growth and metastasis. Podgorski and colleagues demonstrated that lipids from BMAs could fuel prostate tumor cells by upregulating CD36, FABP4, and Perilipin 2, supporting fatty acid transport (84). They also proved that BMAs drive metabolic reprogramming of tumor cells *via* an oxygen-independent mechanism of HIF-1α activation (85). CD36 is a scavenger receptor found on tumor cells, which was shown to be vital for metastasis formation and is currently considered as a potential therapeutic target (86, 87). It can be activated by free fatty acids secreted by BMAs and thus promote cancer growth (88). For prostate cancer bone metastases, researchers were able to show that the oxidative and endoplasmic reticulum (ER) stress pathways activated in BMAs can upregulate the secretion of survivin and heme oxygenase 1 to facilitate tumor cell survival (89). Other studies have demonstrated BMAs to drive FAO in tumor cells embedded in the bone marrow (88). As a parallel research field, bone cancers show similar indications for BMA mediated FAO. In acute monocytic leukemia, BMAs promote the cell survival by facilitating FAO *via* the stress response-associated AMP-activated protein kinase (AMPK). Thus, FAO in BMAs could also be considered as potential therapeutic target in the fight against bone metastases (90). The investigation of adipocyte-rich tissues revealed that ovarian, pancreatic and breast tumor cells can reprogram adipocytes to cancer-associated adipocytes (CAA). This phenotype aids the tumor growth by adipocyte dedifferentiation and release of their lipids, thereby promoting migration, proliferation, survival and chemoresistance (91–93). In this context, Liu et al. were able to show that BMAs can be reprogrammed to support myeloma-induced bone disease (94). Nevertheless, it remains unclear whether BMAs can dedifferentiate into the same tumor-aiding phenotype as found in other adipose tissues. Regarding overall lipid metabolism, researchers have shown that caprylic acid (C8:0) was increased in prostate cancer patients with diagnosed bone metastases (95). These results open a novel research avenue to study the various fatty acid-influenced molecular actions in the BMA-tumor cell interplays.

BMAs may also shape the microenvironment in the bone marrow in aid of tumor cell colonization (96). An expansion of BMAs with age was shown to be associated with a decreased bone mineral density (BMD) in patients (97). Similarly, experiments in mice demonstrated that high-calorie diets induce a shift from osteoblast to adipocyte differentiation, while increasing parameters for osteoclast activity (12). In addition, BMAs can promote osteoclastogenesis by mediation of osteoblast-secreted RANKL (98). These phenotypes are contributing to the severity of BMA-induced tumor burden, by driving osteoclastogenesis and thereby osteolytic lesion formation *via* IL-6 or indirectly *via* CXCL1 and osteopontin (OPN) (11). The CXCL1 and CXCL2 derived from BMAs were shown to promote prostate cancer survival and stiffen the overall tumor immune response (88, 99). Along this line, these chemokines could potentially attract macrophages and attribute to the distinct BMA-altered microenvironment. Studies in omental adipocytes have demonstrated to induce tumor-associated-macrophage polarization by upregulation of *Pparb* expression (88). Further research is needed to define the specific role of monocyte and macrophage sub-populations dependent on the presence of BMAs on the growth of tumor cells. Concerning the B cell lineage, BMAs were shown to overall impair the function of plasma cells compared to other adipocytes in humans (100). However, B cells in bone tumor niches remain an untouched area of research. Nevertheless, BMAs seem to play a pivotal role in the bone niche allowing the tumor cells to move in and grow.

While the fact that BMA-induced direct metabolic alterations on tumor cells poses a relatively wide scientific base, the effect on the metabolism of other resident cells and metastatic tumor progression remains to be largely under-studied. Researchers have shown the importance of metabolism in various tumor microenvironments. Therefore, it stands to reason that BMAs could influence their microenvironment in a similar way. For instance in other murine tumor tissues, it was shown that lipid uptake and FAO in myeloid-derived suppressor cells (MDSCs) facilitate their inhibitory role on anti-tumor T cells and promote tumor cell growth and migration (101, 102). Researchers could also show that tumor regulatory T cells (Tregs) suppress anti-tumor responses. At the same time the lipid metabolism supports the survival and function of Tregs within the hypoxic tumor microenvironment (103, 104). As Tregs also modulate osteoclasts, a potential link should be investigated (105). Moreover, in obese mice, creatine is a key metabolite linking adipocytes and breast tumors (106). Even though it is still unknown whether this fits for BMAs and the skeletal metastatic cells, creatine has been reported to promote the antitumor immune activity of CD8^+^ T cells and reduce the proliferation of subcutaneous tumors (107). Altogether, BMAs and their contribution to bone metastasis growth need to be further elucidated.

## Limitations and Perspectives

Taken together, BMAs are distinct from other adipocyte fat depots, especially in the context of transcriptome, metabolism and functions to direct tumor growth. With novel emerging technologies, more information beneath the tip of the BMA iceberg has been unveiled, and BMAs might be considered as potential target to counteract the bone metastasis in a manner of individual treatment. However, their functions are still puzzling and would require further investigation.

While BMAs in their regulated or constitutive form are better characterized in rodents, this topic remains under-studied in patients. As humans have higher bone marrow adiposity than rodents, the role of BMAs in altering the surrounding environment may differ as well. Studies have shown contradicting conclusions regarding GU and insulin response in rodents versus humans. Here, the lack of receptors or other dissimilarities of the different species have to be taken in account. In humans, the insulin-producing beta cells lack a part of the G-protein-coupled receptor as compared to mice (108). Another dissimilarity observed between the species, was the sex-specific increased rBMA content in female versus male mice (9, 109). Overall, it has to be further elucidated whether the sexual dimorphism in rodent BMAs as well as human white adipose tissue is also reflected on human BMAs (110, 111). It is also important to mention that most of the findings are based on *in vitro* co-culture of diverse tumor cells with isolated bone marrow mesenchymal cells-induced adipocytes or differentiated pre-adipocyte cell lines (e.g. 3T3-L1). However, the reduced lipolytic activity in BMAs *in vivo* could not be recapitulated *in vitro* using these bone marrow mesenchymal stem cells (24, 45). It is always questionable to call *in vitro* differentiated adipocytes real BMAs, as the underlying microenvironmental factors distinguishing them from non-BMAs are lacking. Thus, future studies should rely on the direct *in vivo* evidence between BMAs and tumor cells. Also, the different metabolic or functional manners between BMAs and other adipocyte fat depots in supporting tumor cells colonization should be separately delineated.

Nevertheless, the animal models precisely tracing and locating rBMAs and cBMAs *in vivo* are also what we desperately need in future studies. The animal models will be beneficial for the investigations of BMA subpopulations. Exploit of *Ptrf* knockout initiates the first step towards establishing the rBMAs ablation model (9). Simultaneously, we are also confident that more and preciser markers of these adipocyte subpopulations will emerge in the future due to the utilization of large-scale scRNA-seq analyses. Advanced in-depth analyzing strategies may further help eliminate the contamination of BMAs surrounding cells such as osteoblasts and hematopoietic cells (45).

In addition, future studies need to explore the site-dependent lipid types (rBMAs vs. cBMAs) (9), cellular source and subcellular localization of the altered fatty acids. These investigations will help to quantify the impact of BMAs on local and systemic metabolism, and their function in steady-state or with tumor burden. Thus, the pro-tumor and anti-tumor roles of BMAs will be defined further in the future.

## Author Contributions

XC, YZ and AB designed this review. YL, SC and AG wrote the manuscript. All authors contributed to the article and approved the submitted version.

## Funding

This study was supported by the National Natural Science Foundation of China (81771729, 81971534). This project was also supported by the German Research Foundation (DFG) priority program SPP2084 μBONE. BO-3811/5–1; BO-3811/6–1, Collaborative Research Centre 1181 project A01, Interdisciplinary Center for Clinical Research grant A77 and J76, The European Research Council consolidator grant ODE.

## Conflict of Interest

Author YZ is employed by company Shanghai Huaota Biopharmaceutical Co. Ltd.

The remaining authors declare that the research was conducted in the absence of any commercial or financial relationships that could be construed as a potential conflict of interest.

## Publisher’s Note

All claims expressed in this article are solely those of the authors and do not necessarily represent those of their affiliated organizations, or those of the publisher, the editors and the reviewers. Any product that may be evaluated in this article, or claim that may be made by its manufacturer, is not guaranteed or endorsed by the publisher.
